# Promising Immune Treatment of Advanced Cutaneous Squamous Cell Carcinoma with Cemiplimab—Real-World Experience in the Global SARS-CoV-2 Pandemic

**DOI:** 10.3390/curroncol29100616

**Published:** 2022-10-16

**Authors:** Marta Pabianek, Aleksandra Lesiak, Dariusz Nejc, Łukasz Kuncman, Joanna Narbutt, Małgorzata Skibińska, Magdalena Ciążyńska

**Affiliations:** 1 Department of Proliferative Diseases, Nicolaus Copernicus Multidisciplinary Centre for Oncology and Traumatology, Pabianicka 62, 93-513 Łódź, Poland; 2 Department of Dermatology, Paediatric Dermatology and Oncology Clinic, Medical University of Łódź, 91-347 Łódź, Poland; 3 Department of Surgical Oncology Chair of Oncology, Nicolaus Copernicus Multidisciplinary Centre for Oncology and Traumatology, Medical University in Łódź, 93-513 Łódź, Poland; 4 Department of Radiotherapy, Medical University of Lodz, 90-419 Łódź, Poland

**Keywords:** non melanoma skin cancer, squamous cell carcinoma, immunotherapy, cemiplimab, COVID-19

## Abstract

Cutaneous squamous cell carcinoma (cSCC) is the second most frequent non-melanoma skin cancer. The standard curative treatment is surgical resection, but the treatment of locally advanced and metastatic disease apart from radiotherapy is currently based on cemiplimab. Cemiplimab has demonstrated efficacy in the treatment of advanced and metastatic cSCC in clinical trials, although real-world data are still limited. We present four cases of cSCC, which showed a tremendous response to cemiplimab—one patient achieved complete response and three of them achieved partial response. Immunotherapy with cemiplimab, a recently approved PD1 inhibitor, is an important addition to the cutaneous oncology therapeutic options that may be considered in patients with advanced disease not amenable to surgery or radiotherapy. In all four cases, the patients postponed visits to the doctor because of the fear of SARS-CoV-2 infection or for administrative and organizational reasons declared difficult access to doctors caused by the pandemic.

## 1. Introduction

The incident rate for cutaneous squamous cell carcinoma (cSCC) is increasing significantly. This skin cancer frequently recurs locally after surgical excision and radiotherapy. Even though advanced cutaneous SCCs consist of not many metastatic cases and often patients of locally advanced disease, neoplasms affect also older patients unfit for either radiotherapy or surgery, which could just be considered by systemic therapies. Cemiplimab is a high-affinity human G4 monoclonal antibody that could induce programmed cell death. Cemiplimab, an immune checkpoint inhibitor (ICI), was approved for the treatment of patients with locally advanced or metastatic cutaneous SCC for patients not suitable radiation or surgery [1]. These agents function by attaching to the PD1 receptor on activated immune cells to prevent PD1 from interacting with its ligands, which reduces inhibitory signals and increases the host antitumor response [2]. In the current study, we present the clinical results of four advanced cSCC patients who received cemiplimab treatment during the challenging COVID-19 pandemic period. None of the patients were immunocompromised.

## 2. Case Report 1

**Figure 1:**
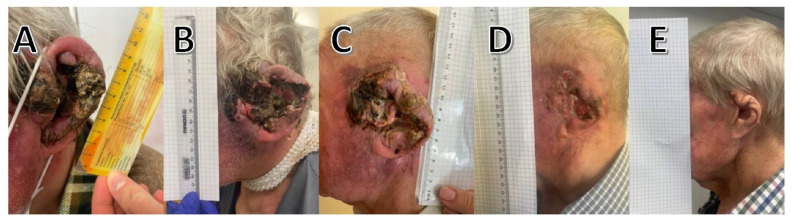
(**A**) Rapidly growing cutaneous mass on the patient’s right ear, visible infiltration of the tissues of the right cheek; (**B**) ulcerative mass prior to therapy; (**C**) healing ulcer base with scarring after one dose (3 weeks) of cemiplimab; (**D**) lesion after 3 doses (9 weeks) of cemiplimab; (**E**) complete response after 18 weeks of cemiplimab (6 cycles).

An 88-year-old man in relatively good condition for his age despite multiple comorbidities, such as arterial hypertension, cardiac failure, and dyslipidemia, was referred to oncology department due to a 1-year history of a rapidly growing cutaneous mass on his right ear (Figure 1A). During this time, the patient did not follow any treatment for this lesion, and because he lived alone, working on a farm, burdened with numerous comorbid diseases, a possible infection with SARS-CoV-2 could cause dramatic consequences for the patient. In the initial period, lesion on the ear underwent a slow, malicious, and extensive evolution, but in the last few months it started to enlarge rapidly. A histological examination of the surgical specimen revealed a well-differentiated infiltrative cutaneous SCC. Physical examination revealed enlarged lymph nodes in the neck and right conglomerate lymph node mass measuring 4 cm at the level of the mandible. Fine-needle biopsy confirmed the presence of cancer metastases in the nodes. Clinically, other smaller but similar skin lesions were localized in the patient’s left cheek, the left ear, and forehead. Moreover, the patient described a loss of hearing in the right ear which had been present for one month. As the tumor intensively infiltrated the surrounding tissues, it entered the right cheek; thus, the lesion was considered inoperable. Three weeks after the first visit to the oncology center, the patient returned to the next visit with the results of the imaging examinations necessary for qualification for systemic treatment. Within 21 days, the lesion enlarged dramatically, and intense tissue proliferation was visible (Figure 1B). Considering the patient’s age, multiple comorbid conditions, exhaustion of options for surgical and radiotherapeutic treatment and following a lengthy conversation with the patient, systemic treatment with cemiplimab was selected as the optimal treatment modality. In May 2021, the patient started receiving cemiplimab (350 mg flat dose every 3 weeks) as part of his immunotherapy. The lesions on the right ear began to heal, progressively thickened, and it stopped oozing soon after the first cycle (Figure 1C). The treatment was well tolerated despite his advanced age and numerous comorbidities, with the exception of occasional intermittent grade 1 pruritus after cycle 6, which was responsive to antihistamines. The patient is currently continuing treatment time. Figure 1D shows the response after 9 weeks of treatment. After 18 weeks (6 cycles) of treatment, a complete response was observed (Figure 1E).

## 3. Case Report 2

**Figure 2:**
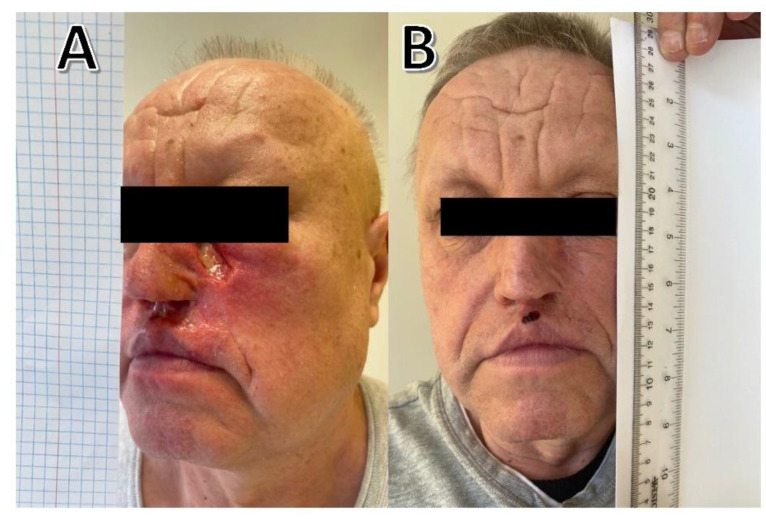
(**A**) Before treatment; (**B**) After 3 administrations of cemiplimab (9 weeks).

A 52-year-old man came in for examination of an incompletely excised cSCC on the left side of his nose that had spread to the nasal septum and left cheek. The tumor had perineural invasion, and pathology had shown that it had extended to the resection margins. Adjuvant radiation therapy would be administered after radical resection, according to multidisciplinary consensus. Due to local relapses, re-operation was performed three times; the final procedure included the resection of part of the left cheek, part of the nasal septum and a fragment of the maxillary sinus. Treatment reduced the tumor mass; however, patient presented to oncology departments with locally advanced tumor in the form of a non-healing ulcer on the right cheek (Figure 2A). Computed tomography (CT) scan was negative for regional or distant metastases. An oncologist recommended immunotherapy. The patient was started on cemiplimab (350 mg every 3 weeks). He experienced wound healing and a remarkable reduction in pain after the first dose, and the ulcer healed completely after three administrations of cemiplimab (9 weeks) (Figure 2B). No toxicity was observed. After seven months of treatment, he continues therapy and demonstrates durable disease control.

## 4. Case Report 3

**Figure 3:**
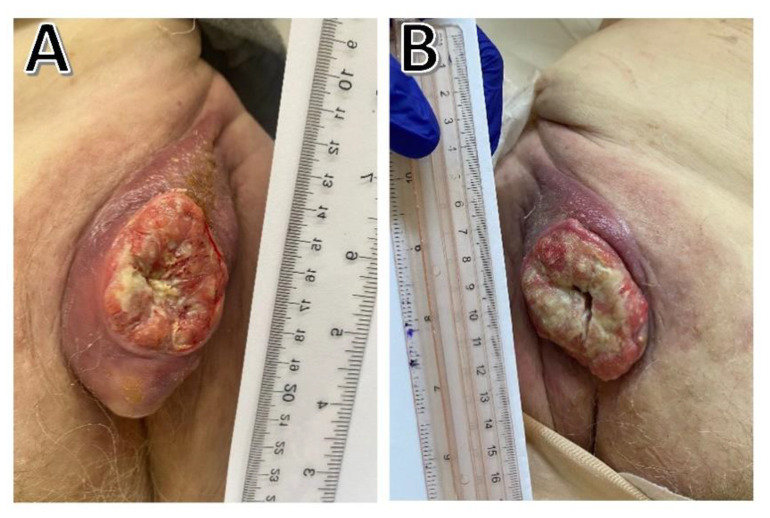
(**A**) Before treatment; (**B**) after 3 administrations of cemiplimab (6 weeks).

A recurrent large tumor was seen in the right inguinal region of a 67-year-old female patient, who was referred to the oncology department for further management. Her past medical history was notable for having hypothyroidism and highly uncontrolled hypertension. In the past, two recurring right inguinal masses were surgically removed (with safe margins) but without the use of postoperative radiotherapy or chemotherapy. Both resected masses had cSCC infiltrative histology with perineural invasion, according to the pathological diagnosis. Physical examination revealed an oval, 5 × 10 cm, painful mass in the right groin that was well-fixed to the underlying tissue and had ulceration of the skin above the alterations (Figure 3A). Using contrast, a computed tomography (CT) scan showed a significant pathologic mass with heterogeneous enhancement at the left groin which remained inseparable from the rectus abdominis and tailors and metastatic to cervical lymph nodes. Due to the extent of the tumor and numerous infiltrations into the surrounding tissues, including the muscle, the tumor was considered nonoperative. Subsequently, the radiation oncology team was consulted—the patient was disqualified from radiation treatment. Therefore, treatment with cemiplimab given intravenously at a dose of 350 mg every 3 weeks was started. The treatment was well tolerated, and the only adverse effect experienced was fatigue, worsening pre-existing hypothyroidism. After two treatments, a massive reduction in the tumor was seen in the CT scan and clinically on the photos (Figure 3B). The treatment is still ongoing.

## 5. Case Report 4

**Figure 4:**
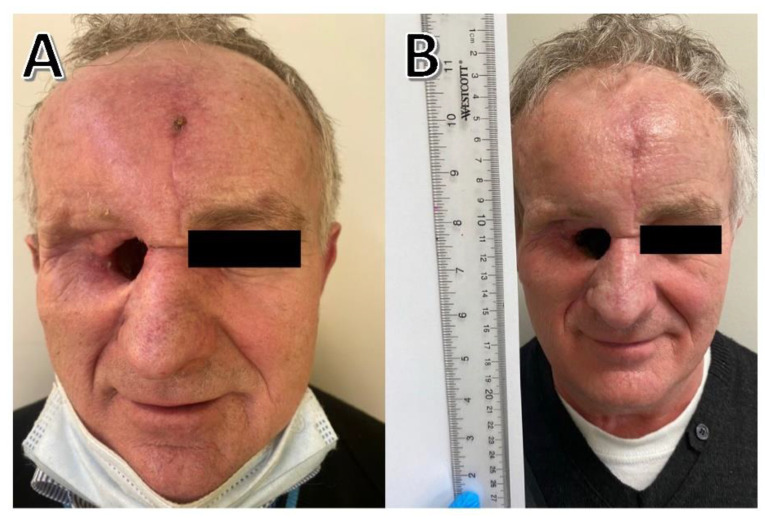
(**A**) Before treatment; small ulceration within the scar where the transplanted skin piece joins the skin of the forehead; (**B**) after 5 weeks of therapy; lesion of the forehead healing.

A 62-year-old male patient with a history of more than 10 keratinocyte carcinomas (cSCCs and basal cell carcinomas) presented to the oncology department to qualification to a systemic treatment. He had undergone multiple resections of cSCCs of the head and neck region, most of which were performed during the preceding 10 years. Seven years before presentation, he had a melanoma on his back, without accompanying ulceration, with a Breslow infiltration depth of 0.4 mm (T1a). At that time, he was treated with wide excision. The patient remained under observation during the next few years. In 2018, he underwent the excision of a rapidly growing tumor within the corner of the right eye. Histological examination of the lesion showed cSCC. Several months after excision, he exhibited a local relapse in the right eyehole area, with a rapidly growing, ulcerated. Due to the COVID-19 pandemic and, as he claims, the difficulty in accessing doctors, the patient delayed the visit and did not control skin lesions during that time. The right orbit lesion was diagnosed as cSCC with infiltrative histology with perineural invasion. The patient was treated by surgical resection with orbital exenteration with following radiotherapy; then, he underwent multiple resections, including skin flap grafting. At the time of presentation, he presented with left temple pain occurring for 1 month and small ulceration within the scar where the transplanted skin piece joins the skin of the forehead (Figure 4A). The wound began as a pruritic papule on his forehead 3 months prior to presentation. A heterogeneously enhancing mass, including the sphenoid bone, rectus muscle, and temporalis muscle, was found in the right orbit site by CT and magnetic resonance imaging, which is compatible with local recurrence. The initial diagnosis was supported by CT with positron emission tomography. The patient was eligible for cemiplimab treatment because all surgical and radiation options had been exhausted. Every three weeks, he received 350 mg of cemiplimab intravenously as treatment. The patient noted total pain relief and healing of the forehead lesion after 5 weeks of therapy (Figure 4B). After 12 weeks, magnetic resonance imaging showed that the pathologic mass in the location of the left orbit had shrunk. The treatment is still ongoing.

## 6. Discussion

With a poor overall survival of only 10.9 months, cutaneous squamous cell carcinoma that is not susceptible to surgery has historically been challenging to treat [3]. Only 20–30% of patients responded to all previous retrospective analyses, including chemotherapy, radiation treatment, and cetuximab [1]. However, growing access to novel oncological therapies including immunotherapy is resulting in the improved prognosis of patients with locally advanced and metastatic cSCC. Immunotherapy with anti-programmed death ligand-1 (PD-1) agents is the standard of care in all current guidelines. Cemiplimab was approved in September 2018 by the U.S. Food and Drug Administration (FDA) anti-PD-1 antibody in inoperative cases that are also not a candidate for radiation [4]. FDA approval was based on clinically meaningful and durable objective response rates (ORR), not on progression-free survival (PFS) or overall survival (OS) data. As presented in the registration study, cemiplimab showed an impressive 50% and 47% response rate for unresectable advanced and metastatic cutaneous squamous cell, respectively [1]. Moreover, Rischin et al., confirmed the sustained substantial clinical activity of cemiplimab during the extended follow-up of outcomes and quality of life analysis [5]. Until recently, chemotherapy for platinum derivatives was the standard. Another alternative therapy in which advances SCC apart from cemiplimab is pembrolizumab (Keytruda). This therapy was recently approved by FDA; however, it is not available in Europe, including Poland.

In accordance with the provisions of the Drug Program of the National Health Fund and with established and generally accepted recommendations RECIST 1.1, a partial response (PR) was defined as a reduction of ≥30% in the clinical and/or radiological diameter, and a complete response (CR) was the complete clinical and radiological disappearance of the tumor. Progression was defined as an increase in the tumoral bulk of ≥20%. All four cSCCs presented responded to cemiplimab treatment. One patient achieved a complete response, and three of them achieved a partial response, which makes these results very promising. All the presented patients continue to receive immunotherapy.

Despite the high clinical activity of cemiplimab, toxicity is a crucial issue that can even lead to treatment discontinuation. The most common adverse effects of cemiplimab in phase 2 trials ae fatigue (41%), diarrhea (27%), and pruritus (27%) [1]. However, we did not report any significant worrying toxicities. Most of them were mild (G1), and none of the patients in our series presented a serious or lethal adverse reaction or discontinued treatment due to AEs. Only one patient suffered from pruritus, who was successfully treated with antihistamines. The frequency and severity of other toxicities, such as diarrhea or fatigue, reported in our patient was low in comparison with the clinical trials. Nevertheless, all patients receiving cemiplimab should be monitored for potential side effects, because these could be severe or even lethal.

It is well established that the previous diagnosis of one skin cancer confers an increased risk of developing one or multiple subsequent cutaneous neoplasm [6]. In one of the presented cases, the patient developed more than 10 previous keratinocyte carcinomas as well as melanoma malignum, which would confirm the concept of field cancerization underlying framework for understanding the pathophysiology and guiding management of multiple cSCCs [7].

Our patients are of particular interest as, to the best of our knowledge, there have been no reports of advanced recurrent and metastatic cutaneous squamous cell carcinoma treated successfully with cemiplimab in Poland before. Moreover, the evidence from real-world studies with advanced and metastatic SCC treated with cemiplimab in central Europe is still limited and mainly consists of single case reports. Recent retrospective studies often include patients treated with several PD-1 inhibitors or are conducted in Spain or in Italy [8,9,10,11], where higher skin phototypes are often present. None of the presented patients were Hispanic or Latino, and all of them had skin phototype I or II. Badami et al. [12] presented the outcomes of 23 patients from the University of Southern Alabama (USA) study who were treated with cemiplimab after its approval in 2018. The outcome results compared with case report data, phase study and our data are presented in Table 1.

This report has some limitations; in fact, it is only four patients’ cases, and the follow-ups were short. However, it illustrates how patients with a rare, advanced disease, which, in most cases, could be cured quickly, through timely excision in initial stages of the disease, neglected due to the fear of infection with SARS-CoV-2, can lead to aggressive, advanced forms, leading to the disfigurement of the patient and lowering their quality of life. The management of cSCC is largely dependent on tumor staging at the time of presentation: curative intent and well prognosis for early stage of disease also palliative intent for rare locally advanced and metastatic disease. Despite the very challenging management of patients with advanced cSCC, cemiplimab has proven to be effective in the treatment of those cases. All four case reports describe a primary lesion that grew out of control due to no contact with the doctor (case 1 and 3) or persistent misdiagnosis by GCP (case 2) or lack of follow-up due to global SARS-CoV-2 pandemic (case 4). Cutaneous SCC rarely progresses to this severity, but we have described this patient and emphasized his rapid healing, and improving all our patients’ quality of life.

## 7. Conclusions

In conclusion, cemiplimab demonstrated its utility in the treatment of advanced and metastatic cSCC in patients at any age with acceptable very good safety profile. We hope that similar patients may also benefit from this remarkably effective novel treatment in Poland.

## Figures and Tables

**Table 1:** Patient characteristics and tumor response summary from available data.

	Our Study	Badami et al. [12]	Zargham et al. [13]	Nelson et al. [14]	Owonikoko et al. [15]	Midgen et al. [16]	Rishin et al. [17]
Patients	4	23	1	1	26	59	56
Male/Female	3/1	20/3	1/0	1/0	21/5	54/5	48/8
Median age	65 (52-88)	74 (39–94)	61	91	73 (55–88)	71 (38–93)	72 (38–96)
Objective Response Rate	100%	78%	100%	100%	50%	48%	41%
Time to First Response [months]	1.4	1.7	0.8	0.8	2.3	1.9	1.8
Complete Response	2 (50%)	8 (35%)	0	1	0	4 (7%)	3 (5%)
Partial Response	2 (50%)	10 (43%)	1	0	13 (50%)	24 (41%)	20 (36%)
Progression Free Survival	n/a	n/a	100%	n/a	89%	81%	47%
Overall Survival at 12 months	n/a	n/a	100%	n/a	53%	n/a	76%
Adverse Events	50%	26%	0%	100%	71.3%	15%	n/a

## Data Availability

No new data were created or analyzed in this study. Data sharing is not applicable to this article.
